# Intensity modulated proton therapy compared to volumetric modulated arc therapy in the irradiation of young female patients with hodgkin’s lymphoma. Assessment of risk of toxicity and secondary cancer induction

**DOI:** 10.1186/s13014-020-1462-2

**Published:** 2020-01-13

**Authors:** Marta Scorsetti, Luca Cozzi, Pierina Navarria, Antonella Fogliata, Alexia Rossi, Davide Franceschini, Fiorenza De Rose, Ciro Franzese, Carmelo Carlo-Stella, Armando Santoro

**Affiliations:** 1 Humanitas Research Hospital and Cancer Center, Radiotherapy and Radiosurgery Department, Via Manzoni 56, 20089, Milan, Rozzano Italy; 2 Department of Biomedical Sciences, Humanitas University, Milan, Rozzano Italy; 3 Diagnostic Imaging Department, Humanitas Research Hospital and Cancer Center, Milan, Rozzano Italy; 4 Oncology & Hematology Department, Humanitas Research Hospital and Cancer Center, Milan, Rozzano Italy

**Keywords:** Intensity modulated proton therapy, VMAT, RapidArc, Lymphoma cancer, NTCP, Seconday cancer risk estimate

## Abstract

**Background:**

To investigate the role of intensity modulated proton therapy (IMPT) compared to volumetric modulated arc therapy (VMAT) for advanced supradiaphragmatic Hodgkin’s lymphoma (HL) in young female patients by assessing dosimetric features and modelling the risk of treatment related complications and radiation-induced secondary malignancies.

**Methods:**

A group of 20 cases (planned according to the involved-site approach) were retrospectively investigated in a comparative planning study. Intensity modulated proton plans (IMPT) were compared to VMAT RapidArc plans (RA). Estimates of toxicity were derived from normal tissue complication probability (NTCP) calculations with either the Lyman or the Poisson models for a number of endpoints. Estimates of the risk of secondary cancer induction were determined for lungs, breasts, esophagus and thyroid. A simple model-based selection strategy was considered as a feasibility proof for the individualized selection of patients suitable for proton therapy.

**Results:**

IMPT and VMAT plans resulted equivalent in terms of target dose distributions, both were capable to ensure high coverage and homogeneity. In terms of conformality, IMPT resulted ~ 10% better than RA plans. Concerning organs at risk, IMPT data presented a systematic improvement (highly significant) over RA for all organs, particularly in the dose range up to 20Gy. This lead to a composite average reduction of NTCP of 2.90 ± 2.24 and a reduction of 0.26 ± 0.22 in the relative risk of cardiac failures. The excess absolute risk per 10,000 patients-years of secondary cancer induction was reduced, with IMPT, of 9.1 ± 3.2, 7.2 ± 3.7 for breast and lung compared to RA. The gain in EAR for thyroid and esophagus was lower than 1. Depending on the arbitrary thresholds applied, the selection rate for proton treatment would have ranged from 5 to 75%.

**Conclusion:**

In relation to young female patients with advanced supradiaphragmatic HL, IMPT can in general offer improved dose-volume sparing of organs at risk leading to an anticipated lower risk of early or late treatment related toxicities. This would reflect also in significantly lower risk of secondary malignancies induction compared to advanced photon based techniques. Depending on the selection thresholds and with all the limits of a non-validated and very basic model, it can be anticipated that a significant fraction of patients might be suitable for proton treatments if all the risk factors would be accounted for.

## Background

The role of proton therapy in the radiation treatment of Hodgkn’s lymphoma (HL) patients as been investigated by many groups and various reviews and consensus publications summarized the status of the art [1–3].

The HL subcommittee of the particle therapy cooperative group (PTCOG) published in 2017 an evidence-based critical review [2] aiming to summarize the rationale for proton therapy based on i) the late morbidity data/models, ii) the dosimetric literature showing the potential of protons over photons and iii) the limited clinical literature. In this review, seconday cancers were identified as the primary cause of death among the long term survivors with particular concern for breast and lung. Cardiovascular morbidity/mortality was then identified as the primary cause of nonmalignant deaths/disease followed by pulmonary late effects (pneumonitis) and endocrinopathies. Dosimetric data provided evidence of the expected superiority of proton based plans over photon based plans for a large number of organs at risk while providing equivalent or superior potential for target coverage. Concerning the clinical studies, no proton-related high grade toxicity (late or acute) were reported with the caveat of sometimes short follow-up in the analysed cohorts. In general, the use of more conformal proton techniques was not associated to marginal recurrences with the promis to guarantee the excellent cure rates derived from photon series. As a recommendation, the PTCOG subcommittee advised that proton therapy should be reasonably considered for HL treatments with the recommendation for the development of a model-based approach for the selection of patients suitable for proton therapy.

More recently, the International lymphoma radiation oncology group published a consensus recommendation in the form of guidelines for proton therapy in adults with mediastinal HL [3]. The report recommended the need to demonstrate (with calculations from comparative plans) the benefit expected from protons. The authors recommended a documented medical necessity (including estimates of the risks for radiogenic late effects) and raised awareness about the increased complexity of proton planning, with special attention to the uncertainty management. The presence of uncertainty deriving from the trade-off between technique and anatomy (range and calibration uncertainties) suggested to treat patients under deep inspiration breath hold to improve organs at risk sparing and to apply robust optimization methods to mitigate the technical uncertainties. The guidelines included a valuable summary to evidence-based acceptable dose-volume constraints for heart structures, breasts, lungs and thyroid which were considered as an input also in the present study.

Due to the favorable physical properties of protons, the dose distributions of intensity modulated proton plans should inherently lead to an increased sparing of organs at risk and to the possibility of dose escalation compared to photon based treatments (when clinically appropriate). Nevertheless, in a resource-limited world, it is necessary to validate the added value of the anticipated dosimetric advantages for radiation toxicity, eventually in synergy with the possibility to prevent long term radiation induced effects (e.g. carcinogenesis). Simple methods based on the investigation of physical dose distributions or simple complication models, mostly at planning level, have been discussed but could only provide general indications and not individualized selection criteria [4–8]. The best approach to validation would be the execution of properly sized and defined clinical trials but this could be limited by ethical considerations, the time needed to obtain the results and the costs of execution. Alternative methods include the development of model-based validation strategies. This concept is founded on the assumption that predictive models for radiation-induced side effects can be developed and applied to estimate the risk of normal tissue complications (on multivariate level) and of other complications like secondary cancer induction for each patient. Based on the model predictions it would be possible to select patients for a given technique among a group of competitive solutions. There is a growing consensus toward the application of this strategies and in some countries (e.g. the Netherlands) this approach will be a pre-requisite for enrollment of patients into proton treatments [9, 10]. The practical implementation of the model-based approach requires well designed data collection in order to fit the dose-effect relationships into multivariate models and to-date only few examples of validated models exist [11–14]. Considering the long life expectancy of the HL patients, the models used for treatment technique selection should incorporate also the risk of secondary cancer induction and the number of years lost due to any kind of late effects [15]. Up to-date no similar tools are available for HL.

Based on the paucity of evidence about this topic, the primary aim of this study was to investigate, at planning level, the relative merit of IMPT versus VMAT for young female patients with supradiaphragmatic HL. In particular, the focus was set to: i) the expected gain in a number of selected dose-volume metrics; ii) the estimation of the risk of complications for relevant organs at risk, and iii) the estimation of the risk of secondary malignancy induction for breast, lung or thyroid. Plans were designed with robust optimization methods to account for the recommendations from [3].

The secondary aim was, in the absence of validated model-based criteria for the assignment of patients to IMPT or VMAT treatments, to appraise with a simplified approach the frequency of indications to proton treatment. The selection criteria were based on simple thresholds applied to composite risks of toxicity and/or secondary cancer induction. Scope of this was to perform a very preliminar verification of the selection power under the assumption that not all patients might necessarily require proton treatments.

## Materials and methods

### Patients selection, contouring and dose prescription

A cohort of 20 young female patients with stage IIA-B supradiaphragmatic HL were selected for this retrospective planning study. The cases were selected from most recent patients in the clinical database fulfilling the criteria above. The selection spanned back to 2016. All patients datasets were used for proton and photon planning.

The involved site radiation therapy concept was applied for the study. The clinical target volume (CTV) was defined as the involved lymph nodes region at diagnosis. The planning target volume (PTV) was generated adding an isotropic margin of 8 mm to the CTV (cropped 4 mm inside the skin surface). The following organs at risk (OAR) were segmented and considered: the breasts and the lungs, heart, the left anterior descending coronary (LAD), the left ventricle (LV), the esophagus, the thyroid and the spinal cord. For the LAD a planning risk volume was considered adding a margin of 4 mm. LAD and LV structures were contoured with the support of a senior diagnostic radiologist on the conventional planning CT datasets. All segmentations were executed on standard contrast-free planning CT acquired in free breathing mode.

The dose prescription was 30Gy in 15 daily fractions as in the clinical routine. For the planning comparison purposes, all the plans were normalized to 100% as the mean dose to the CTV. The dose-volume planning aims used for the optimization and assessment are listed in detail in Tables 1 and 2 for each structure. For the OARs the values were derived from [3].

**Table 1:** Normal tissue complication parameters for the different models and endpoints applied in the study. The uncertainty values reported within brackets are derived from the original publications and correspond to 1 standard deviation

		Lyman model parameters			Poisson-LQ model parameters			
Organ	Endpoint	TD50, Gy	n	m	D50,Gy	∆	Seriality	Ref
Heart	Death	–	–	–	52.4 [49.1–57.1]	1.30 [1.06–1.66]	1 [−1.27]	Gagliardi [16]
Lung	Pneumonitis	–	–	–	34.0 [nr]	0.90 [nr]	0.06 [nr]	Seppenwoolde [17]
	Pneumonitis grade ≥ 2	30.8 [27–38]	0.99 [0.6–1.8]	0.37 [0.30–0.46]	–	–	–	Seppenwoolde [17]
	Symptomatic or radiographic pneumonitis	21.9 [13.2–30.6]	0.80 [0.32–1.28]	0.37 [0.17–0.57]	–	–	–	Moiseenko [18]
	Symptomatic fibrosis	25.0 [17.0–32.0]	0.85 [0.39–1.31]	0.15 [0.09–0.21]	–	–	–	Moiseenko [18]
Esophagus	Esophagitis grade ≥ 2	51.0 [40.0–63.0]	0.44 [0.25–0.79]	0.32 [0.25–0.43]	–	–	–	Chapet [19]

*nr* not reported

**Table 2:** Summary of the planning objectives and average results (uncertainty expressed as 1 standard deviation) for the clinical target volume (CTV) and for the planning target volume (PTV)

Parameter	Objective	RA	IMPT	p
CTV				
Volume: 303 ± 226 Range: [50–927] cm^3^				
Mean [Gy]	30	30.0 ± 0.0	30.0 ± 0.0	
D_95%_ [Gy]	> 29.4 (98%)	29.6 ± 0.1	29.6 ± 0.1	0.3
D_98%_ [Gy]	> 25.5 (95%)	29.4 ± 0.1	29.4 ± 0.2	0.5
D_1%_ [Gy]	< 32.1 (107%)	30.9 ± 0.2	31.0 ± 0.2	0.1
St. Dev [Gy]	Minimize	0.3 ± 0.1	0.4 ± 0.1	0.01
HI	Minimize	0.04 ± 0.01	0.05 ± 0.01	0.1
PTV				
Volume: 639 ± 370 Range: [117–1456] cm^3^				
Mean [Gy]	30	29.9 ± 0.1	29.9 ± 0.1	0.3
D_95%_ [Gy]	> 28.5 (95%)	28.7 ± 0.3	28.8 ± .03	0.2
D_98%_ [Gy]	> 27.0 (90%)	28.0 ± 0.4	28.1 ± .04	0.5
D_2%_ [Gy]	< 32.1 (107%)	31.1 ± 0.2	31.4 ± 0.3	0.01
St. Dev [Gy]	Minimize	0.6 ± 0.1	0.7 ± 0.2	0.08
HI	Minimize	0.07 ± 0.02	0.06 ± 0.01	0.07

*RA* RapidArc, *IMPT* Intensity modulated proton therapy, *D*_*x%*_ dose received by at least (maximum) x% of the volume, *HI* (D_5_-D_95_)/D_mean_

### Photon planning

The photon plans were designed and optimized according to the volumetric modulated arc therapy in its RapidArc (RA). Flattening filter free photon beams (beam quality of 6MV) from a TrueBeam linear accelerator (Varian Medical Systems, Palo Alto, USA) were used for the study. Optimization was performed using the Eclipse treatment planning system Photon Optimiser algorithm and the final dose calculation with the Acuros-XB engine with a calculation grid of 2.5 mm (version 15.5). All plans were optimised according to a class solution consisting of two full arcs with collimator angle set to 10–350°. An additional third arc with collimator angle set to 90° was added if necessary to improve dose distributions. This latter was eventually split into 2 sub-arcs overlapping along the cranio-caudal axis if the field length would be longer than 15 cm.

### Proton planning

The ProBeam proton system (Varian Medical systems, Palo Alto, USA) was used as a source of beam data for the optimization and calculation of intensity modulated proton therapy (IMPT) plans using the beam spot scanning technique. The dose distribution optimization was performed using the nonlinear universal Proton Optimiser (NUPO, v15.5) [20] while for the final dose calculation, the Proton Convolution Superposition algorithm (v15.5) was used using a grid size of 2.5 mm and a constant relative biological effectiveness RBE of 1.1.

All patients were planned with a standardised class solution with one anterior field and two posterior-oblique beams arranged with individualized gantry angles according to the target position aiming to minimize the healthy tissue involvement [5].

A robust optimization technique was applied in order to account for setup and range uncertainties considering ±4 mm shifts in the isocentre along each axis and ± 3% in beam range. The robust optimization was run to result in the solution which minimizes the trade-offs due to the applied uncertainties to the dose-volume constraints in the cost function as discussed in [21]; robust plans were optimised by applying the uncertainties to the CTVs.

### Quantitative assessment of dose-volume metrics

The mean dose and a variety of V_x_ and D_x_ parameters (V_x_ represents the volume receiving at least an x level of dose (in % or in Gy) and D_x_ is the minimum dose that covers an x fraction of volume (in % or in cm^3^) [22] were derived from the dose volume histograms (DVH) and used as quantitative metrics.

For the target, the homogeneity index (HI) was scored to measure the variance of the dose. HI was defined as HI = (D_5%_-D_95%_)/D_mean_. The dose conformality was scored with the Conformity Index, CI95%, defined as the ratio between the patient volume receiving at least 95% of the prescribed dose and the PTV volume. The average DVHs were computed, for each structure and each cohort, with a dose binning resolution of 0.02Gy. Proton doses are reported in Cobalt equivalent Gy (corrected for the 1.1 RBE factor).

The significance of the observed differences was determined by means of the Wilcoxon matched-paired signed-rank test which is appropriate to assess the significance of the discrepancies between two matched pair of variables, not normally distributed in small samples. The threshold for statistical significance was set at *p* < 0.05. The SPSS package version 22 (IBM Corporation) was used for the study.

### Modelling the risk of toxicity and secondary cancer induction

Normal tissue complication probabilities (NTCP) for selected endpoints were estimated for the lungs (pneumonitis), the esophagus (esophagitis) and the whole heart (death). The lungs were jointly considered as a single structure. Two models were used for the computation: the Lyman-Kutcher-Burman [23] and the seriality (Poisson-LQ) model [24]. The parameters used for the computation are summarized in Table 1 together with associated uncertainties as derived from the original publications. Data were retrived from: Gagliardi [16] for cardiac mortality (from the analysis of breast patients treated with photons); Seppenvolde [17] for pneumonitis (from data of breast cancer, malignant lymphoma and non-small-cell lung cancer patients); Moiseenko [18] for symptomatic pneumonitis (from malignant thymoma patients); Chapet [19] for esophagitis (from non-small-cell lung cancer patients). For calculation, the biological evaluation model implemented in Elcipse was used. Other endpoints, included in first instance but leading to null NTCP estimation were not included in this report.

The relative risk (RR) estimation for LAD and LV failure/disease was performed according to the linear model proposed by van Nimwegen [25, 26] for Hodgkin lymphoma patients (following the observations of Darby [27] for breast cancer patients) who correlated coronary heart disease to the mean dose to the heart. The excess relative risk was fixed to 7.4 and 9.0% per Gy, respectively for LAD and LV. The choice of applying the van Nimwegen model using as input the mean dose to the LAD is somehow arbitrary in the absence of definitive data on sensitivity of substructures but is consistent with other similar investigations such as, e.g., Levis et al. [28].

Composite values for NTCP and RR predictions were also computed and reported. For NTCP, the composite prediction was defined as the sum of NTCP for esophagitis, pneumonitis (all three estimates) and cardiac mortality (Composite 1). For RR the composite resulted in the sum of the individual RR for LAD and left ventricule (Composite 2). The composite estimate shall be considered as a worst case scenario where all the possible toxicities concur simultaneously with the same weighting factor.

Concerning the risk of secondary malignancy induction, the excess absolute risk (EAR) for a whole specific organ (*org)* was estimated according to the methods described in [29]. In brief, it is expressed by:

$$ {EAR}^{org}=\mu\ \frac{1}{V_T}\sum \limits_iV\left({D}_i\right) RED\left({D}_i\right) $$

where *V*_*T*_ is the total organ volume. The sum is over all the bins of the differential DVH, *V(D*_*i*_*)* is the absolute volume receiving a dose *D*_*i*_. ∆ is the slope of cancer induction based on the atomic bomb survivors’ data [30] corrected for the age distribution. The values used in this analysis were: 4.98, 3.78, 3.2 and 0.4 for breast, lung, esophagus and thyroid. *RED(D)* is the dose response, which has been modeled using different approaches to fit the Hodgkin’s patient’s data group [30]. The organ equivalent dose (OED): $OED=\frac{1}{V_T}\sum \limits_iV\left({D}_i\right) RED\left({D}_i\right)$ was introduced as the dose in Gray, which, when uniformly distributed across the organ, causes the same radiation-induced cancer incidence.

Different models were published to calculate OED. In the present study, the so-called full model was adopted [31].

$$ OED=\frac{1}{V_T}\sum \limits_iV\left({D}_i\right)\ \frac{e^{-{\alpha}^{\prime }{D}_i}}{\alpha^{\prime }R}\ \left(1-2R+{R}^2{e}^{\alpha^{\prime }{D}_i}-{\left(1-R\right)}^2\ {e}^{-\frac{\alpha^{\prime }R}{1-R}\ {D}_i}\right) $$

Where *R* is the parameter accounting for repopulation and/or repair and models the ability of the tissue to recover between two dose fractions (*R = 0* means no recovery, *R = 1* full recovery). This model fully includes all the biological aspects of cell killing, repopulation/repair, and fractionation. The used values for *R* were 0.62, 0.83, 0.846, 0 for breast, lung, esophagus and thyroid cancer induction, respectively [30–32]. In the case of thyroid, dose-response curves show no repair and are bell-shaped [32]. ${\alpha}^{\prime }=\alpha +\beta\ d=\alpha +\beta \frac{D}{N}$ where *N* is the number of fractions of the treatment. The used values for α, derived from the same sources, were: 0.067Gy^− 1^, 0.022Gy^− 1^,0.46 Gy^− 1^ and 0.0318Gy^− 1^ respectively [Schneider_1]. α/β is the common LQ parameter: α/β = 3 Gy.

Also for the EAR a composite value was defined as the sum of the EAR per each organ with the same worst case scenario approach used for NTCP and RR.

### Model based selection of the appropriate technique

An attempt to determine selection criteria for the potential assignment of patients to either photon based or proton based treatments, a simple model based approach was derived from NTCP, RR and EAR estimates.

In the absence of validated models for lymphoma patients, arbitrary thresholds were defined for each of the composite estimates as follows: NTCP composite: thresholds set at either 8% or 5%; RR composite: threshold set to 0.25 or 0.10. For EAR the threshold on the composite value was set to either 15 or 10.

Given the definition of the composite estimates, this model based selection aims to detect the maximum possible benefit of one technique over the other in the rare case of all toxicities manifesting simultaneously. More sophisticated models, based on clinical data, should be developed in the case of clinical use.

The model based selection would then require that a patient would have been assigned to the IMPT treatment according to possible 3 scenarios (from more selective to more inclusive):

Strong criteria: estimates concomitantly in excess of all the three higher thresholds;

Intermediate criteria: estimates concomitantly in excess of all the three lower thresholds;

Weak criteria: estimates in excess of at least 1 high or 2 low thresholds.

It is absolutely obvious that, given the small sample size of the study, the approach suggested here cannot be considered more than a feasibility aiming to pave the path for more appropriate and comprehensive models in view of any clinical applicability [9–15].

## Results

### Dosimetric comparison

**Fig. 1:**
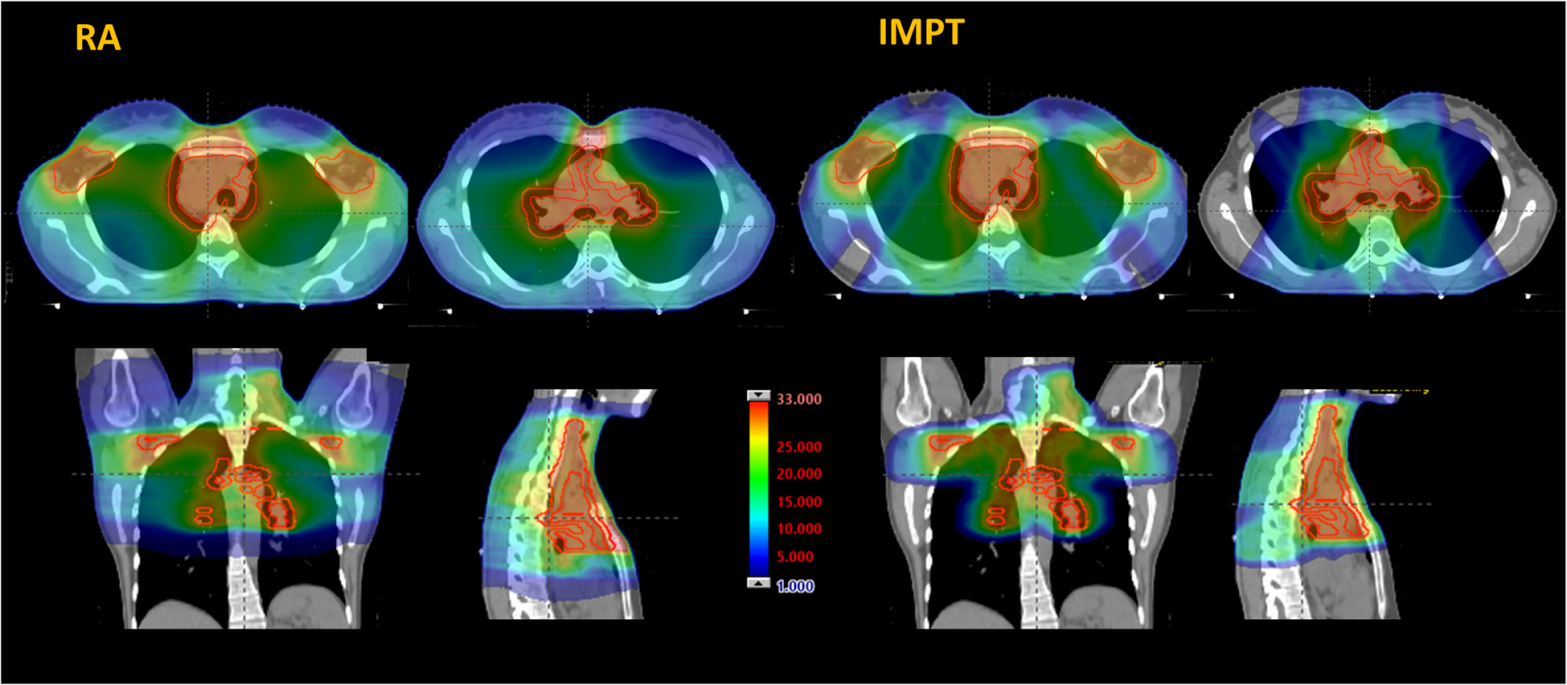
An example of the dose distribution for the proton and photon plans in axial, coronal and sagittal planes. The colorwash was set from 1 to 33Gy

**Fig. 2:**
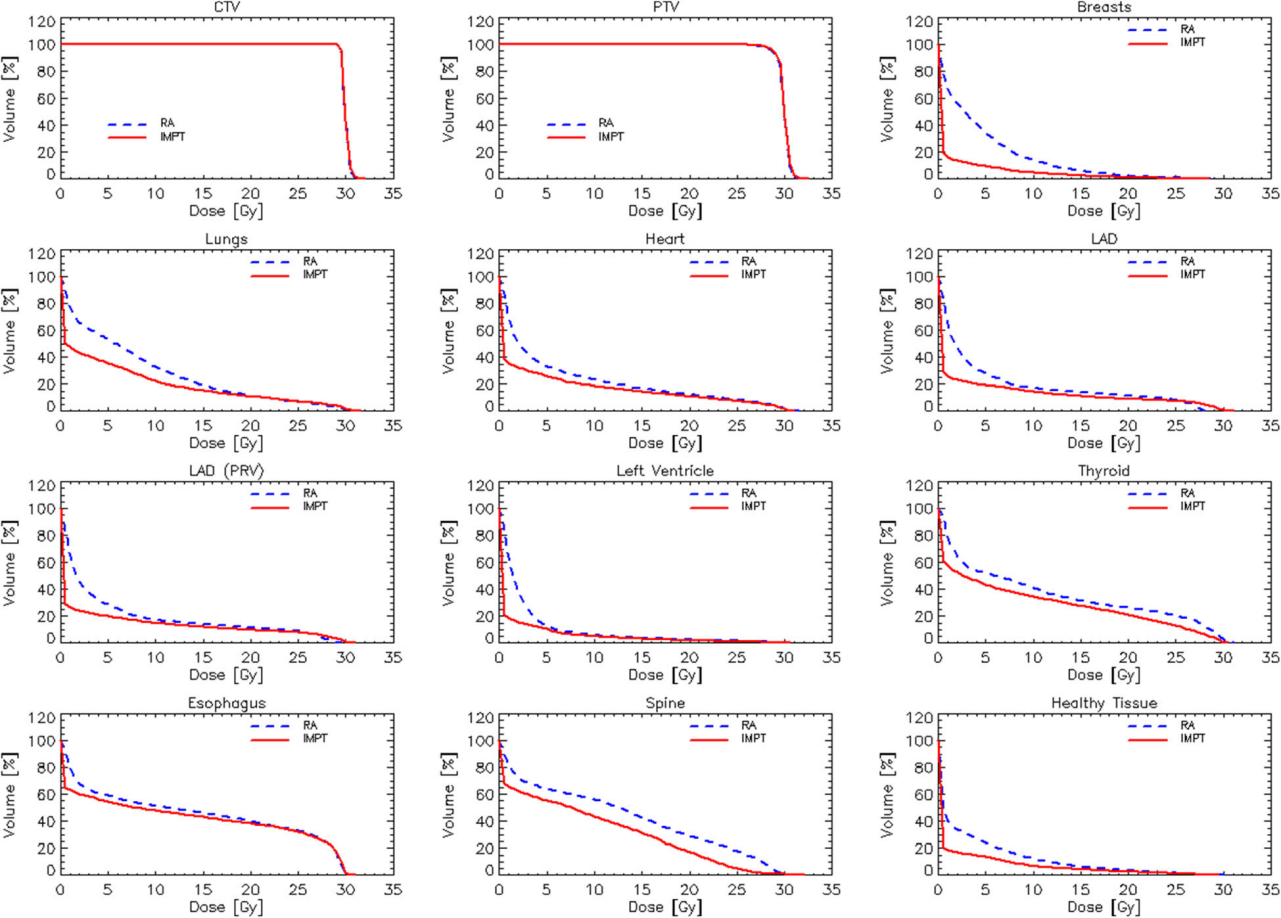
Average dose volume histograms for the target volumes and the main organs at risk investigated. Solid lines correspond to robust intensity modulated protons and dashed lines correspond to volumetric modulated arc photon plans

Fig. 1 illustrates the dose distributions for one representative case for the RA and IMPT plans in two axial and the coronal and sagittal planes. The colorwash was set from 1 to 33Gy. Fig. 2 presents the average dose volume histograms for the two techniques for the CTV, the PTV and the main organs at risk considered in the study. In front to an equivalence for the target volumes, the IMPT robust plans presented an average large sparing effect over the dose range 0-15Gy for most of the organs and over the entire range for the spine and thyroid. In the case of the esophagus the sparing effect of protons is less remarkable since this OAR is either partially included or very proximal to the target. For LV protons showed the sparing effect up to 5Gy while for higher doses no average difference was seen with respect to photons.

This qualitative overview reflects the quantitative analysis of the DVHs summarized in Table 2 for CTV and PTV and in Table 3 for the OARs. Regarding target volumes the only statistically significant differences were marked for the standard deviation in the case of the CTV (0.1Gy difference) and for the near-to-maximum dose D_2%_ for the PTV (0.3Gy, i.e. 1% difference). In both cases the clinical relevance of such a difference seems to be negligible. Contrarily to the target volumes, for all the OARs statistically significant differences were observed for most of the relevant dose-volume metrics and near to significant (*p* < 0.1) for the remaining.

**Table 3:** Summary of the planning objectives and average results (uncertainty expressed as 1 standard deviation) for the main organs at risk investigated in the study

Parameter	Objective	RA	IMPT	p
Breasts				
Volume: 1808 ± 964 Range: [516–3886] cm^3^				
Mean [Gy]	<4Gy	4.7 ± 2.5	1.4 ± 1.2	< 0.001
D_1%_ [Gy]	Minimize	20.6 ± 5.0	17.5 ± 7.0	0.03
Lungs				
Volume: 2260 ± 530 Range: [1183–3313] cm^3^				
Mean [Gy]	< 10 Gy	8.3 ± 2.6	5.9 ± 2.6	< 0.001
V_20Gy_ [%]	< 20%	11.6 ± 6.5	11.0 ± 6.6	< 0.001
V_5Gy_ [%]	< 55–60%	53.5 ± 15.4	35.8 ± 13.5	< 0.001
Lungs-PTV				
Volume: 2118 ± 534 Range: [1034–3188] cm^3^				
Mean [Gy]	< 10 Gy	7.4 ± 2.2	4.9 ± 2.0	< 0.001
V_20Gy_ [%]	< 20%	7.8 ± 4.1	7.1 ± 4.2	< 0.001
V_5Gy_ [%]	< 55–60%	51.7 ± 14.9	33.2 ± 12.5	< 0.001
Heart				
Volume: 557 ± 110 Range [403–728] cm^3^				
Mean [Gy]	Minimize	6.6 ± 3.8	4.9 ± 3.6	< 0.001
D_1%_ [Gy]	Minimize	25.9 ± 10.0	25.3 ± 11.2	0.06
LAD				
Volume: 3.1 ± 0.6 Range: [2.3–4.4] cm^3^				
Mean [Gy]	< 5 Gy	5.6 ± 4.5	3.8 ± 5.4	0.002
D_1%_ [Gy]	Minimize	16.1v9.9	12.9 ± 11.9	0.08
LAD (prv)				
Volume: 11.2 ± 1.7 Range: [8.3–14.5] cm^3^				
Mean [Gy]	< 5 Gy	5.7 ± 4.6	4.0 ± 5.4	< 0.001
D_1%_ [Gy]	Minimize	17.1 ± 10.0	13.6 ± 12.2	0.03
Left Ventricle				
Volume: 117 ± 48 Range [114–320] cm^3^				
Mean [Gy]	< 5 Gy	2.9 ± 2.2	1.6 ± 2.0	< 0.001
D_1%_ [Gy]	Minimize	13.6 ± 11.6	11.5 ± 12.5	0.03
Thyroid				
Volume: 9.7 ± 4.3 Range: [6.3–20.9] cm^3^				
Mean [Gy]	Minimize	10.8 ± 9.0	8.6 ± 7.7	< 0.001
V_25Gy_ [Gy]	< 62.5 Gy	20.6 ± 25.3	13.0 ± 14.5	0.03
Spine				
Volume: 38.6 ± 12.7 Range: [17.3–66.8] cm^3^				
D_1%_ [Gy]	Minimize	26.5 ± 5.5	22.7 ± 5.6	0.06
Esophagus				
Volume: 27.3 ± 6.2 Range: [18.8 ± 39.2] cm^3^				
Mean [Gy]	Minimize	13.9 ± 4.8	12.8 ± 5.9	0.01
D_1%_ [Gy]	Minimize	29.3 ± 2.2	29.1 ± 2.6	0.1
Healthy Tissue				
V10Gy [%]	Minimize	12.3 ± 5.8	7.0 ± 3.4	0.001
CI_95%_	1.0	1.2 ± 0.1	1.1 ± 0.1	0.002

*RA* RapidArc, *IMPT* Intensity modulated proton therapy, *Prv* planning risk volume, *D*_*x%*_ dose received by at least (maximum) x% of the volume, *V*_*xGy*_ volume receiving x Gy

When the analysis is restricted to the metrics having explicit planning aims, IMPT plans respected the constraints in all cases while RA plans resulted slightly sub-optimal for the mean dose to the breasts, to the LAD (0.7Gy higher than the aim in both cases).

The dose conformity was similar for RA and IMPT (~ 10% better for IMPT) but, as inherently expected, the dose bath resulted largely reduced with Protons. The V_10Gy_ metric resulted 75% higher for RA than for IMPT.

### Toxicity and secondary cancer risk estimates

Table 4 reports the the RR estimates for the LAD and LV as well as the NTCP estimates for the two techniques and the various endpoints considered in the analysis and for the composite estimates. The uncertainties reported are relative to the inter-patient variance. The uncertainty on the NTCP predictions due to the uncertainty on the model’s parameters are not included here but were determined to be of the same order of the discrepancy observed between photons and protons.

**Table 4:** Estimates of the normal tissue complication probabilities according to the various models applied and endpoints considered. Results are shown as averages (with uncertainty expressed as 1 standard deviation) and median value (second line). The *p* value is relative to the Wilcoxon signed rank paired test

Organ	Model	Endpoint	RA	IMPT	∆(RA-IMPT)	p
LAD	RR	Failure	1.41 ± 0.34	1.28 ± 0.39	0.13 ± 0.15	0.001
			1.28	1.16		
Left Ventricle	RR	Failure	1.26 ± 0.20	1.13 ± 0.18	0.13 ± 0.10	< 0.001
			1.20	1.02		
Heart	NTCP	Death (LQ)	0.16 ± 0.16	0.15 ± 0.13	0.02 ± 0.04	0.03
			0.14	0.12	0.01	
Lungs	NTCP	Pneum LQ	0.30 ± 0.37	0.14 ± 0.20	0.16 ± 0.21	< 0.001
			0.17	0.05	0.09	
	NTCP	Pneum LKB	1.98 ± 1.04	1.34 ± 0.74	0.64 ± 0.47	< 0.001
			1.77	1.09	0.55	
	NTCP	SymPneum LKB	4.61 ± 2.98	3.19 ± 2.27	1.42 ± 1.13	< 0.001
			3.84	2.36	1.09	
	NTCP	SymFibr LKB	3.26 ± 1.79	2.75 ± 1.72	0.51 ± 0.45	< 0.001
			2.75	2.22	0.38	
Esophagus	NTCP	Esophagitis	2.61 ± 1.49	2.47 ± 1.52	0.15 ± 0.27	0.04
			2.47	2.35	0.14	
Composite 1	NTCP	–	12.92 ± 7.09	10.02 ± 5.83	2.90 ± 2.24	< 0.001
			11.04	8.17	2.24	
Composite 2	RR	–	2.68 ± 0.51	2.41 ± 0.55	0.26 ± 0.22	< 0.001
			2.46	2.18	0.22	

*RR* relative risk, *NTCP* normal tissue complication probability, *Pneum LQ* Pneumonitis with Poisson-LQ model, *Pneum LKB* Pneumonitis with Lyman model, *SymPneum LKB* Symptomatic or radiographic pneumonitis ≤6 months with Lyman model, *SymFibr LKB* Symptomatic or radiographic fibrosis > 6 months with Lyman Model

Based on these results, the findings from proton plans are suggestive of a remarkable sparing effect with a highly significant average reduction of the RR of 0.13 for both LAD and LV.

In the case of NTCP, for all the endpoints the IMPT plans resulted in highly significant reduction of the risk or toxicity compared to RA although in this case the average risk for each individual endpoint would be relatively small, lower then 5%. This statement is correct under the working assumption that the model-related uncertainties cancel out in the computation of the NTCP difference (∆(RA-IMPT)). Otherwise, the reported differences, at individual level, would not be significant. The average composite gain with protons in NTCP risk resulted < 3%, and < 0.3 for RR.

The analysis of the EAR (expressed in cases per 10,000 patient-years) showed a systematic and highly significant expected benefit for IMPT with respect to RA as summarized in Table 5. In particular, the average EAR gain (defined as the EAR for photons – the EAR for protons) resulted 9.1 and 7.2 for the breasts and the lungs, respectively. The estimated EAR gain did not reach 1 case per 10,000 patient-years for the thyroid and the oesophagus so, although statistically significant, this cannot be considered of clinical relevance.

**Table 5:** Excess absolute risk (EAR) (per 10,000 patient-years) of secondary cancer induction estimated with the full model. Results are expressed as average (with 1 standard deviation as uncertainty) and median values

	RA	IMPT	∆RA-IMPT)	*p*
Breast	12.6 ± 4.9	3.5 ± 3.3	9.1 ± 3.2	< 0.001
	12.2	2.4	9.0	
Lung	22.6 ± 8.0	15.3 ± 8.0	7.2 ± 3.7	< 0.001
	22.8	15.8	7.4	
Thyroid	1.7 ± 1.0	1.3 ± 1.1	0.3 ± 0.4	< 0.001
	1.8	1.5	0.2	
Esophagus	3.4 ± 0.5	2.6 ± 0.6	0.8 ± 0.3	< 0.001
	3.4	2.5	0.8	
Composite	40.2 ± 10.6	22.7 ± 8.2	17.5 ± 5.4	< 0.001
	40.3	22.5	16.5	

*RA* RapidArc Volumetric modulated arc therapy, *IMPT* intensity modulated proton therapy with robust optimization

### Model based selection of the appropriate technique

Based on the arbitrary thresholds, the model based selection of patients simulated in this study would have lead to: 1 (5%) patient only eligible to proton therapy in the case of the strong, 4 (20%) patients in the case of the intermediate and 15 (75%) patients in the case of application of the weak criteria.

## Discussion

The present study compared IMPT and RA plans for 20 HL patients. Prior to discuss the findings of the study, it is fundamental to acknowledge the limit of the sample size. Although it is common practice of in-silico planning studies to select small groups of patients, it is obvious that this approach does not allot to accurately capture all the risk factors due to the large uncertainties caused by contouring and organ motion. The role of a planning study is to identify the potential of a technique (compared to other references) and to pave the path to well-defined clinical investigations. The analysis of dose-volume metrics demonstrated a systematic incremental sparing of all OARs achievable with protons over photons. These results are in line with other planning evaluations [4–6]. Existing data from literature confirm the low toxicity profile expected from protons. Hoppe [33] reported the early outcome for HL patients treated by consolidative proton therapy. In a cohort of 138 patients (pediatric and adults), the 3-year relapse free survival was 92% with no grade 3 radiation-related toxicities. Similarly, König [7] reported about the treatment of 20 patients. Homogeneity index resulted 1.04, (coherently with the data from our study) with a 1-2Gy reduction observed in the mean dose to the breast compared to photon based intensity modulation plans (~ 3.3Gy in our evaluation) and 3-4Gy for the dose to the heart (~ 1.7 in our group). After a median follow up time of 32 months, the relapse free survival was 95.5% and no toxicity greater than 1–2 was observed. These results are a direct confirmation of the low incidence of severe complications in HL treatments but, in the absence of long follow-up time it does not fully clarify the long term morbidity/mortality risks.

The dosimetric potential advantage is translated into an anticipated reduction of risk of toxicity. Any estimate of normal tissue complication is based on set of parameters derived from the analysis of clinical dataset, frequently small and possibly obtained from treatments not strictly consistent with those under investigation. This relatively arbitrary choice of parameters shall be considered if absolute estimates are to be appraised. In addition, all model parameters are affected by inherent uncertainties (often large) which are reflected into the predictions. This can be seen as an implicit bias in the calculations which can be mitigated expressing the results in relative terms, as ratios or differences as we did in this study. This working hypothesis is used in the present study. Otherwise, due to the large uncertainty on the parameters concurring to the computation of NTCP (see Table 1), the discrepancy between the NTCP predictions for photon and proton plans, would be of the same order of the model-related uncertainty cancelling the significance of the differences at a patient-per-patient level. This issue should be mitigated only by an improved determination, with small variance, of the parameters in the NTCP model; a fact of major relevance for model-based selection of the treatment to select for the patients. The use of non-parametric tests to determine the potential significance of the discrepancies can accounts for the small sample sizes. This was evaluated with NTCP for the lungs, the whole heart and the oesophagus with different endpoints and different models using evidence-based input parameters for the calculations. A more recent approach was adopted for the estimation of the relative risk of morbidity for the heart substructures, namely the left ventricular chamber and the left anterior descending coronary. In this case the Nimwegen-Darby models [25–27] were applied and also in this case treatments with IMPT have the potential to reduce the risk on average. Levis [28] applied the same models for RR in a comparison among two variants of VMAT. They found that RR for the LV was 1.3 ± 0.6 while RR for the coronaries (all together) was 2.0 ± 0.6 in comparison with the 1.26 ± 0.20 and 1.41 ± 0.34 for the RA plans in our study. This demonstrates the consistency of VMAT data among different institutions with different planning environments. It is important to notice, as limiting factors, that the van Nimvegen model vas developed correlating the coronary disease to the mean heart dose and not to the sub-structure dose. In addition, the data presented in those studies were derived from photon treatments and therefore the models might not be strictly applicable also to protons. In the absence of definitive published data, the assumptions made, although arbitrary, would allow an appraisal of the relative risks.

The RR estimates for LAD and LV (and heart substructures in general) are biased by at least 2 major limits: i) the identification of these structures onto planning CT datasets and ii) the motion induced by heart beat. The use of the planning risk volume concept is an effective mitigation for the motion uncertainties of the coronaries (as applied in this study). The possibility to identify the heart sub-structures on normal (contrast free) treatment planning CT scans is instead quite limited. As a consequence of the challenges in segmenting the LAD on contrast-free planning CT (as in our study) it is obvious that the results presented shall be associated to an uncertainty which is potentially severe and certainly hard to quantify. A possible mitigation of both issues might be the advisable use of cardiac scanning (with the need of registration against the planning CT and the associated extra costs). Alternatively, Levis [28] proposed to use atlas-based contouring guidelines (as defined by Feng [34]). This approach might facilitate the taks, particularly if dedicated cardiac scanning would not be available.

The calculations for the excess absolute risk of secondary cancer induction were performed by means of the so-called full model as described in the methods. This accounts for repair, repopulation and fractionation mechanisms. For the thyroid, epidemiologic data suggest the absence of any repair mechanism (as shown as an example by Bhatti [35]) so the parameter R is set to 0. Mathematically, the limit for R approaching R of the EAR function reduces it to the bell shaped form. In practice, EAR resulted quite large for breast and lung and strongly reduced with IMPT. EAR for lung was in absolute terms large also for protons (15.3 ± 8.0) because of the extensive dosimetric involvement of these organs for supradiaphragmatic HL patients. On the contrary, the thyroid and the oesophagus resulted in lower absolute EARs (in the range from 1 to 3.5 in average) with modest improvement offered by protons. In summary, lung and breast confirmed to be the structures at highest risk and, for (young) female patients, the risk reduction (a sparing of ~ 72% from the RA level) expected from protons is likely the strongest benefit derived from the data analysed.

Given the large variability of target locations and extension, although all classified as supradiaphragmatic cases, the level of protection of each OAR, in particular the heart sub-structures, presents a large variance in all considered metrics (dose-volume parameters, NTCP, RR and EAR) confirming that, a case by case assessment of the appropriate treatment technique should be performed with a model-based methodology.

The absence of validated multivariate models applicable for HL patients prevented to implement the selection strategies suggested by Langendijk and realized by Rwigema or Cheng [9–11, 13] for head and neck or Prayongrat [14] for liver. This is a clear need and intensive investigations shall be performed in the near future in this area to provide reliable model. A simple attempt to mimic the process was introduce in this study. In practice thresholds with different selection power (weaker or stronger) were applied to composite risk estimates (NTCP, RR and EAR) to determine scenarios of decreasing selectivity. Without the claim of generalization, we demonstrated that, even with quite relaxed criteria, not all patients would result eligible for proton therapy (up to 75% in the most inclusive scenario) while, given the anyhow high quality of VMAT plans, if highly restrictive criteria would be applied, only a minority of the cases (eventually of the order of 5%) might result eligible. It is obvious that this speculation should be considered with caution but it is suggestive/confirmative of the fact that: i) selection is needed in a resource-limited health economic environment and ii) also HL patients can benefit from proton therapy, particularly for the groups at highest risk as young females (or pediatrics).

The IMPT plans used for this analysis were obtained by means of a robust optimization engine using 3% uncertainty in the range calibration and 4 mm in the isocenter position. The thresholds are somehow arbitrary but realistic from routine clinical practice. The use of robust optimization is coherent with the guidelines of the International Lymphoma Radiation Oncology group [3]. Although not fully consolidated in clinical practice, the use of robust optimization should be seen as an essential ingredient of the planning receipt.

Even if dosimetric studies shown that pencil beam scanning can be superior to other techniques [36] the same studies demonstrated that the quality of plans is sensitive to motion/positioning related issues which can be accounted for or mitigated by means of the robust optimization methods.

Motion due to respiration can be further mitigated by respiratory gating, and in this perspective, deep inspiration breath hold (DIBH, a practice largely consolidated in photon treatments in the breast or chest districts) can provide additional benefit in terms or dose reduction to various OARs. The use of DIBH is also part of the guidelines from the international lymphoma group. Everett [6] showed how DIBH could improve lung dosimetry but might had little impact on cardiac involvement in mediastinal lymphoma treatments. Similarly Edvardsson [8] demonstrated the benefit for lung and the limited value for breasts (with large variations depending on the individual case for the heart structures). In the present study, the CT datasets used for the planning were acquired in free breathing (due to the current clinical practice for HL in our institute) and it can be anticipated that all the dosimetric findings for lung might be further improved according to the above indications. Nevertheless the benefit would be consistently present in both RA and IMPT plans and therefore [5] the relative comparison between the two approaches would not be substantially different. It is clear that in the case of future clinical investigations, DIBH should be included as a prerequisite. The heartbeat is a challenging factor that could affect the dose delivery uncertainty. There are today no tools allowing any sort of gating or tracking following the heartbeat, contrarily to what happen with the respiration. In the supradiaphragmatic region, heartbeat changes the anatomy with a frequency higher than, and not syncronyzed with the dose delivery, generating an unforeseen and unintended interplay effect, especially for spot delivery in IMPT treatments.

## Conclusion

In relation to young female patients with advanced supradiaphragmatic HL, IMPT can offer an improved dose-volume sparing of organs at risk leading to an anticipated lower risk of early or late treatment related toxicities. This would reflect also in significantly lower risk of secondary malignancies induction compared to advanced photon based techniques. Depending on the selection thresholds and with all the limits of a non-validated and vary basic model, it can be anticipated that a significant fraction of patients might be suitable for proton treatments if all the risk factors would be accounted for.

## Data Availability

The datasets used and analysed during the current study are available from the corresponding author.

## Abbreviations

CTV: Clinical target volume
EAR: Excess absolute risk
HI: Homogeneity index
HL: Hodgkin’s lymphoma
IMPT: Intensity modulated proton therapy
LAD: Left anterior descending coronary
LQ: Linear-quadratic
LV: Left ventricle
MV: Megavoltage
NTCP: Normal tissue complication probability
NUPO: Nonlinear universal proton optimizer
OAR: Organ at risk
OED: Organ equivalent dose
PTCOG: Particle therapy cooperative group
PTV: Planning target volume
RA: RapidArc
RBE: Relative biological effectiveness
RR: Relative risk
VMAT: Volumetric modulated arc therapy
